# Overcoming the Digital Divide for Older Patients With Respiratory Disease: Focus Group Study

**DOI:** 10.2196/44028

**Published:** 2023-10-03

**Authors:** Esther Metting, Sanne van Luenen, Anna-Jetske Baron, Anthony Tran, Stijn van Duinhoven, Niels H Chavannes, Maud Hevink, Jos Lüers, Janwillem Kocks

**Affiliations:** 1 Data Science Center in Health University Medical Center Groningen University of Groningen Groningen Netherlands; 2 Department of Primary and Elderly Care University Medical Center Groningen University of Groningen Groningen Netherlands; 3 Department of Operations Faculty of Economics and Business University of Groningen Groningen Netherlands; 4 Department of Public Health and Primary Care Leiden University Medical Center Leiden Netherlands; 5 Groningen Research Institute for Asthma and COPD University Medical Center Groningen University of Groningen Groningen Netherlands; 6 Department of General Practice and Elderly Care Medicine University Medical Center Groningen University of Groningen Groningen Netherlands; 7 Department of Medicine University of Amsterdam Amsterdam Netherlands; 8 Leiden University Leiden Netherlands; 9 Department of Psychiatry and Neuropsychology School of Mental Health and Neuroscience, Alzheimer Centre Limburg Maastricht University Maastricht Netherlands; 10 Farmacologica, Nictiz Groningen Netherlands; 11 General Practitioners Research Institute University of Groningen Groningen Netherlands; 12 Observational and Pragmatic Research Institute Singapore Singapore; 13 Department of Pulmonology University Medical Center Groningen University of Groningen Groningen Netherlands

**Keywords:** elderly people, usability, asthma, chronic obstructive pulmonary disease, website, navigation

## Abstract

**Background:**

The need for and adoption of eHealth programs are growing worldwide. However, access can be limited among patients with low socioeconomic backgrounds, often resulting in a so-called “digital divide” due to a mismatch between eHealth and target populations that can gain benefit. This digital divide can result in unsuccessful eHealth implementations, which is of critical importance to health care.

**Objective:**

This study evaluated the opinions of elderly patients with asthma and chronic obstructive pulmonary disease (COPD) about an existing pharmacy-based personalized patient web portal that provides medication overview and information on associated diagnoses. The aim was to obtain insights on the common barriers of elderly people when using health-related websites, which can help to improve accessibility.

**Methods:**

This was a cross-sectional qualitative study of a patient panel of the Groningen Research Institute for Asthma and COPD in primary care. Participants were required to be older than 55 years, be Dutch speaking, have no prior experience with the study website, and be diagnosed with a chronic respiratory illness. Two focus groups were created, and they completed a 45-minute session for testing the website and a 120-minute session for semistructured interviews. The focus group sessions were recorded, transcribed verbatim, and analyzed by content analysis.

**Results:**

We enrolled 11 patients (9 women) with a mean age of 66 (SD 9) years. Of these, 5 had asthma, 3 had COPD, 2 had asthma-COPD overlap syndrome, and 1 had bronchiectasis. Participants were generally positive about the website, especially the areas providing disease-related information and the medication overview. They appreciated that the website would enable them to share this information with other health care providers. However, some difficulties were reported with navigation, such as opening a new tab, and others reported that the layout of the website was difficult either because of visual impairments or problems with navigation. It was also felt that monitoring would only be relevant if it is also checked by health care professionals as part of a treatment plan. Participants mentioned few privacy or safety concerns.

**Conclusions:**

It is feasible to develop websites for elderly patients; however, developers must take the specific needs and limitations of elderly people into account (eg, navigation problems, poor vision, or poor hand-eye coordination). The provision of information appears to be the most important aspect of the website, and as such, we should endeavor to ensure that the layout and navigation remain basic and accessible. Patients are only motivated to use self-management applications if they are an integrated part of their treatment. The usability of the website can be improved by including older people during development and by implementing design features that can improve accessibility in this group.

## Introduction

Population aging has been reported to be associated with a significant increase in the number of people with chronic noncommunicable diseases (NCDs) that adversely affect daily life [1]. Cardiovascular disease, diabetes, cancer, and chronic respiratory disease are the most common of these, typically requiring daily self-management by patients to reduce symptoms and prevent deterioration, and medication adherence, trigger avoidance, and maintaining a healthy lifestyle are important for these efforts. Health care providers can support patients to improve these skills by shifting from a paternalistic approach to a collaborative approach that encourages active patient engagement.

Patients today are better educated and have easy access to health-related information through the internet. Over recent years, digital health or eHealth has come to the fore as a means of supporting patients and health care providers in the management of chronic illnesses. According to the World Health Organization, eHealth refers to “cost-effective and secure use of information and communications technologies in support of health and health-related fields” [2]. This broad definition covers the myriad of health applications that are currently available, ranging from digital measurement instruments (eg, online questionnaires) to smartphone apps or websites. National and international governmental organizations have promoted the use of digital health by setting ambitious goals and providing grant support for policymakers and developers in this field. For example, in the Netherlands, health care organizations, insurance companies, and the government published a leading report regarding the future of Dutch health care [3]. In this report, partners agreed that care should be provided digitally when possible. Digital and technological innovations are essential to ensure access to health care for Dutch citizens [3]. Presently, the internet is used by 88% (2021) of all people in the Netherlands, with 52% (2021) of those older than 75 years using it more than once a week [4]. Other data show that 76% (2022) of Dutch citizens use the internet to search for health-related information [5]. However, many patients with a low socioeconomic status and elderly patients are difficult to reach via digital health, despite potentially having the most to benefit. This is because digital literacy is strongly related with socioeconomic status and age. The mismatch between increased use of digital health and the inability of the target population to use it is the so-called “digital divide,” and it can result in unsuccessful digital health implementations [6].

Elderly people and people with a low socioeconomic status need additional support to improve their digital literacy to overcome the digital divide. This type of literacy has been defined as “the ability to seek, find, understand, and appreciate health information from electronic sources and apply the knowledge gained to address or solve a health problem” [7]. There are several important barriers to digital health access, including the number of chronic conditions (negatively associated with internet use), low income (inability to pay for internet access or computer equipment), low confidence and limited experience (not growing up with the internet or computers), low education levels, and being housebound [7,8]. All of these barriers are related to poor digital health literacy. However, there has been a shift in the proportion of internet users among older people in the Netherlands over the past 10 years. In 2012, it was reported that 52% of those aged 65-75 years and 16% of those aged >75 years used an internet modem almost daily, and by 2022, the proportions had increased to 84% and 61%, respectively [9,10]. These data show that a high number of people, including elderly people, have access to the internet. This makes the use of digital health possible for a broader population. Elderly people are therefore important health technology consumers, and it is important to include them in consumer informatics research.

The development of digital health for elderly people has been termed “gerontechnology” [11], and it has the potential to support managing age-related problems and difficulties [12]. Indeed, digital health interventions could prove effective in enhancing quality of life and social participation, reducing social isolation, and enabling independent living [6-8,10]. By empowering patients, digital health may improve not only self-management [6,13], but also a person’s lifestyle and physical activity levels [14]. Having access to a website that provides information about medical conditions can enhance a patient’s knowledge of their disease, which is essential for good self-management [13]. Despite the importance of stakeholder evaluation for successful implementation [15] and despite older people having a valuable contribution to make [6,12,16], elderly patients are often excluded from active participation in technology development. Considering evidence that elderly groups use technology differently from younger groups [16], this can lead to unusable digital health apps.

Diseases like asthma and chronic obstructive pulmonary disease (COPD) are typical NCDs, affecting 5.5% and 4.6% of elderly Dutch residents, respectively. Good self-management of both has been shown to reduce the numbers of hospital admissions, general practitioner (GP) consultations, and oral steroid and antibiotic prescriptions [17]. Education about self-management should include not only disease-specific information and technical skills but also problem-solving and communication skills, with the latter focusing on patients being able to have meaningful discussions with health care providers [18]. However, patients with asthma and COPD tend to have poor educational attainments and socioeconomic statuses, especially those with severe disease [19]. If correctly targeted, personalized digital health delivered via web portals or apps could help to improve self-management in this large patient group.

In this study, we aimed to evaluate the opinions of elderly patients with chronic respiratory diseases (focusing on asthma and COPD) about an existing pharmacy-based personalized patient web portal (PWP) providing medication overview and information on their conditions. The personal health record is comparable with that used in PWPs elsewhere and with that used by other health care websites, so we anticipate that the results of this study can be generalized to other digital health settings. The findings of the study will be helpful to obtain insights into the common barriers that need to be addressed in order to improve the accessibility of websites for elderly people.

## Methods

### Study Design and Participants

This cross-sectional qualitative study has been reported according to the Consolidated Criteria for Reporting Qualitative Research (COREQ) guidelines [20]. Participants were recruited via the primary care patient panel of the Groningen Research Institute for Asthma and COPD (GRIAC), using the following inclusion criteria: age older than 55 years, Dutch speaking, no prior experience of the study website, and diagnosis of a chronic respiratory illness. Emails were sent to ask if the prospective participant would evaluate a website to be used by patients with asthma and COPD. All participants were asked to sign an informed consent form before participation.

### Focus Groups

#### Structure

All interviews were led by EIM (a female psychologist/epidemiologist) and AJB (a female behavioral scientist/research assistant). EIM has considerable experience both moderating focus groups and evaluating digital health apps with patients, and she knew 4 of the participants from previous focus group studies. All patients were offered a free lunch during the focus groups. At the end of each meeting, the interviewer summarized the discussion, checked if the summary information was correct, and asked whether there were any issues that had not been discussed. EIM was also involved in coding the data along with MH (research assistant, primary care), AT (medical master’s student), and SvL (behavioral scientist). All had experience with qualitative data analysis.

Participants were divided into 2 groups for separate meetings that were conducted as follows: a 45-minute computer task, a 15-minute break, and a 90-minute focus group discussion with a 10-minute break at the midpoint. Each participant was first placed at a desktop computer, given access to a website, and asked to complete 9 tasks that were written down on individual forms (Table 1). Participants were free to ask the researchers questions, but they could not engage with other participants during the tasks. After the computer tasks, participants were provided a 15-minute break before the focus group discussions, which lasted 2 hours and included a 10-minute break at the midpoint. 

**Table 1:** Website tasks that participants were asked to execute on a desktop computer.

Task	Explanation of the task
Go to the website	The researcher supplies the website’s URL.
Log in to the website	The researcher supplies a username and password.
Locate medication side effects	The website has information of an example patient with COPD^a^, detailing his diagnosis and medication. Patients were asked to look up the side effects of his medication.
Identify what to do if side effects are experienced	The website provides information about side effects for all prescribed drugs, with information about how to manage each.
Check when to take inhaled medication	The website has information about when medication should be taken for the example patient.
Check if it is possible to quit medication suddenly	The website offers information about the consequences of suddenly stopping the prescribed medication.
Identify the currently prescribed medication	A medication overview is supplied for the example patient with COPD.
Locate the video “How do the lungs work?”	Information about asthma and COPD is provided on the website via links to several lung-related videos that are hosted on other domains (eg, the Dutch Lung Foundation).
Go to the webpage of the Lung Foundation	Links to several reliable websites are available, including a link to the Dutch Lung Foundation.
Identify the pollen outlook for tomorrow (task for asthmatics)	The website has information about pollen and includes a forecast of the next day’s pollen outlook in different areas.

^a^COPD: chronic obstructive pulmonary disease.

We used a website produced by a Dutch pharmacy organization, which includes information about the patients’ diseases, medications, and medication side effects, as well as relevant self-management advice. This site was chosen because it is a good example of a standard PWP provided by health care organizations. However, we cannot offer further details because the provider wishes to remain anonymous.

#### Content 

Semistructured interview schedules were adopted to guide the focus group sessions. Although the computer tasks were not recorded, researchers did note any topics or issues that emerged when performing tasks, and these notes were also used to guide the focus group interviews. All focus group interviews were video and audio recorded. 

Participants were encouraged to share their opinions and to discuss them with other participants. The focus group discussions included 6 main topics, but these focused broadly on the website itself (their first impression, the layout, and the logging in process) and the information provided (about medication, asthma and COPD, and smoking). We used screenshots of the website when discussing specific areas and topics. To obtain unbiased first impressions and to facilitate discussions about the website, we asked participants to write their thoughts on sticky notes first.

### Data Analysis

The focus group recordings were transcribed verbatim and analyzed by content analysis. NVivo version 12 (QSR International) was used as the coding program by 2 pairs of reviewers who coded the data independently (group 1: MH and EIM; group 2: AT and SvL). After the initial coding procedure, the groups had several meetings in order to reach consensus regarding the final coding tree. The qualitative results were based on the output of focus group discussions. The variations of opinions in the groups are presented and illustrated by citations of participants. In this type of research, it is not possible to provide quantitative output measurements (eg, means and numbers). IBM SPSS version 25 (IBM Corp) was used to perform the descriptive analysis. All descriptive data are presented as absolute numbers or as means and standard deviations. 

### Ethical Considerations

This study was performed according to the ethical guidelines of our academic university. Ethical approval was obtained from the medical ethics committee of the University Medical Center Groningen (number M19.224943). All participants were informed by the primary researcher and signed an informed consent form. The sessions were audio recorded, and the data were stored on a secured server of the University Medical Center Groningen. The audio data were removed from the voice recorder after transcription. The questionnaire data were completely anonymous. The audio data were transcribed, and all retrievable information was removed from the transcripts (eg, names). All analyses (in SPSS and NVivo) were performed in a secured working environment of the University Medical Center Groningen.

## Results

### Overview of Participants

We enrolled 11 patients with an average age of 66 (SD 9) years (2 men and 9 women). Of these 11 patients, 5 had asthma, 3 had COPD, 2 had asthma-COPD overlap syndrome, and 1 had bronchiectasis. The cohort was divided into 2 groups, and the comparison of their features is presented in Table 2. All meetings took place in December 2018 at the University Medical Center Groningen.

**Table 2:** Overview of patient characteristics by focus group.

Characteristic			Total		Group 1		Group 2
Age (years), mean (SD)			66.0 (9.0)		71.3 (7.8)		59.6 (7.1)
Gender (female/male), n			9/2		5/1		4/1
**Diagnosis, n **							
	Asthma	5		1		4	
	COPD^a^	3		2		1	
	Asthma-COPD overlap	2		2		0	
	Bronchiectasis	1		1		0	

^a^COPD: chronic obstructive pulmonary disease.

### Overview of the Coding Results 

Table 3 presents an overview of the 21 codes that we identified from the participants’ comments, together with a description of the data included per code. All codes were related to the website, its content, and potentially superfluous or missing data. Specific comments made by participants are covered in the subsequent text. 

**Table 3:** Overview of the coding results based on all comments about the website by participants.

Codes	Description (based on all comments relating to the code)
General impression	Comments about using the whole website, including the first impression of the website.
Side effects overview	Comments about the side effects overview (eg, both finding it and the layout of the page).
Login	Comments about the login process and alternatives.
Medication overview	Comments about the medication overview and its use.
Navigation	Comments about website navigation (eg, searching/finding certain information and linking to external websites).
Missing information	Comments about information categories participants felt needed to be added.
BMI calculation tool	Comments about the BMI calculation tool (eg, name and use of the tool).
Separation of asthma and COPD^a^	Comments about the separation of information on smoking and BMI for asthma and COPD on the website.
Physical activity	Comments about exercise and the different types practiced.
Effects of asthma and COPD	Personal experiences of how asthma or COPD affects daily function.
Medication	All medication-related comments.
Information about side effects	Comments about the various sources of information for medication side effects related to COPD and asthma (eg, medication leaflets), including the use of those sources.
Inhaled medication	Comments about using inhaled medications and the importance of information about the inhaler technique.
Medication use	Comments about experiences with any medication used (excluding inhaled medication).
Medication access by caregivers	Comments about access to medication by other care workers.
Regularity of medication intake	Comments about the importance of regular or timely medication use.
Irrelevant information	Comments about information that participants felt did not need to be included on the website.
Smoking	Comments about smoking (eg, information, association with symptoms, and quitting).
Smoking cessation	Specific comments about why quitting smoking is important and about the information provided on quitting.
Questionnaire	Comments about familiarity with and the use of questionnaires for monitoring COPD and asthma.
Caregivers	Comments about visits and information from caregivers (eg, nurses, general practitioners, pulmonologists, rehabilitation doctors, and pharmacists).

^a^COPD: chronic obstructive pulmonary disease.

### First Impression of the Entire Website 

Participants were generally satisfied with the website. Some participants stated that they would use the website for information about their disease, but others thought that there was too much text. Nevertheless, those who expressed positive opinions welcomed the fact that information was gathered on 1 webpage. The videos and links to relevant websites were also appreciated. 

For myself of when I am short of breath. Or, yes for general information. 

Some participants recommended that developers should use a larger font and improve the contrast of the website to help with readability (eg, “I need my reading glasses”). Most participants did not know that they could enlarge the letters in the browser. According to the participants, the website needs to be accessible for patients with poor vision. 

Participants also felt that there was scope to expand the website to include information about other pulmonary illnesses, such as bronchitis, and the role of inhaled allergens. Others also commented that the website may benefit from including some information about the social impact of asthma and COPD. 

And I also think, how do you deal with loneliness […]? 

It was also felt that the website should not be too complicated.* *

They always try to put as much as possible in it, but they should first make sure its correct. 

#### Navigation

Most participants had difficulties finding the information that they needed for the tasks, with only 1 participant considering the website to be “clear” and others using terms like “redundant” and “too much” to describe the amount of information on the website. Several participants wanted the information to be more “concise” to improve the time needed to find necessary information. Consistent with this, many sentences were considered to be too long and too complicated. 

Because you currently spend a lot of time searching.

I lost my orientation.

During the tasks, participants were asked to open a video, which was opened in a new browser page. Participants reported trying to click the “back” arrow to return to the prior page, but this did not work because they were on a new page. The researchers had to help most participants return to the original website during the tasks. These issues were mentioned during the focus group discussion. 

I always have the tendency to click on the arrow to go back.

I was looking for information, about the video probably, about the working mechanism of the lungs. I wanted to go back but I couldn’t.

Some participants found that the multiple different text blocks gave the website a disorganized appearance. They reported needing to scroll to see all information on the website and that it was unclear when they left the website (eg, a new page opened). 

I left the website without noticing it. 

#### Logging in

Participants could login using a username and password, but it was not easy to find the correct button to login. Most participants were also unhappy about needing to use a password because every website requires one, and they stated it can be difficult to remember them all. 

I hate passwords so much! 

That makes me mad. Then you need another for that, then another for that.

They suggested using the Dutch authorization system used by the government or using 2-factor authentication with an SMS text message code. However, 2 participants did not have a mobile phone and would have been unable to use this method. 

I just have email and nothing else. 

#### Distinction Between Asthma and COPD

Participants were required to distinguish between asthma and COPD on the website, and this produced different lifestyle advice. Participants accepted the need for this because the illnesses are fundamentally different, but they did feel that there was some information overlap, especially regarding physical activity and smoking, which can be provided to patients with either asthma or COPD. 

### Medication

#### Medication Overview

Participants reported that the medication overview was very clear and easy to follow. They stated that it might be helpful to be able to print a PDF version of the list to present to other health care professionals. Several participants with other medical conditions reported having experienced suboptimal communication between health care providers. 

What is difficult … it’s not merged .... Once, when I was hospitalized, I needed to have a conversation with the pharmacist; he came but said to me that he didn’t have any information about my medication. 

The medication overview was also of use to participants, particularly where it provided an overview of past exacerbations and when a course of antibiotics or prednisolone could be stopped. 

I actually have to, if I have an exacerbation ... I should actually keep track of my prednisone use from that and that [date] until that and that date.

#### Side Effects

The information about side effects was considered to be clear, but some participants only got to the relevant page by accident. Participants also appreciated the link provided to the Dutch Information Center for medication side effects (LAREB), where they could report side effects. It was not clear to everyone that this link opened a new web page. 

Some participants read all information because they wanted to know what to be vigilant for and whether the medication might interfere with other conditions. Some participants only reviewed patient information leaflets and side effects when they first received new drugs. 

I have a heart condition and ... if a physician or specialist doesn’t know what I cannot have, well … I always want to check for myself. 

By contrast, other participants did not want to know about the side effects. Several participants mentioned that they never looked at these leaflets because they would get worried about the side effects. 

I throw it all away. 

I never considered this important … [in medication] … because it doesn’t make me happy. 

It makes me scared. 

#### Inhaler Technique

Good inhaler technique was considered important and was often mentioned during focus group sessions. Participants were aware that poor inhaler technique was a highly relevant issue in respiratory health care. The videos on the website were well received, though participants did feel that they cannot substitute face-to-face instructions. One participant thought that the videos were overly brief. Some participants also noted that instructions on cleaning the inhaler should be included on the website. 

How do you do that? Do you use a paper towel? Or not? How do you keep your spacer anti-static …? 

I always fall into that trap. That you neglect things or that you are puffing too quickly, aren't you? And that you don't take your time to do it right. 

#### Medication Apps on the Website

The website offered medication reminder and repeat prescription services. The former included the option to set reminders about when to take medications, and participants felt that this could be useful in improving adherence to their regimens. The repeat prescription service was considered very useful, but participants also reported that it could be improved by adding automated warnings to indicate when a new prescription is needed. 

### Self-management

#### Consequences of Asthma and COPD

Many of the participants reported having other chronic illnesses that required them to visit physiotherapists, dieticians, pulmonologists, primary care physicians, and pharmacists. Several participants mentioned that there were issues with the use of antibiotics or prednisolone during exacerbations, that complaints about second-hand smoke and air pollution due to heaters can lead to misunderstandings, and that pet allergies could lead to social isolation because others did not understand the need for avoidance. In addition, most participants complained about having a lack of energy due to their illness. 

Loneliness is the worst thing there is ..., that damn loneliness, I have had to deal with it all my life. With asthma, not [going] to birthdays [and not going to this or that]. 

And that is tiring when I have to talk and that also costs energy. And then with me that is very bad; but yes, they can do nothing for me. 

#### Symptom Questionnaires

In the future, the website will offer the ability to complete symptom questionnaires to monitor the status of asthma or COPD. Most patients have experience with these types of questionnaires because they use them with health care professionals as a routine part of their disease assessment. The opinions about adding these questionnaires to the website varied significantly, with some participants thinking they would never use them and others stating that they would be very useful. 

I don't need it. No, if I have complaints, the GP will refer me to the pulmonologist.

... you have a good clear questionnaire, which you have to fill in if you use certain medication or you feel unwell for a while, whatever it is … that you could put it down in a very clear way … and possibly for [healthcare professionals to] have access to it

### Lifestyle

#### Smoking and Physical Activity

Some participants commented that information about smoking was difficult to find on the website, and most participants stressed that there was a need to add information about physical activity. It was also observed that information about physical activity and smoking cessation was presented differently for asthma and COPD, but that it was probably appropriate to merge this information because of its relevance to both patient groups. More specifically, only patients with asthma were informed about the interaction between smoking and medications, but it was agreed that such information should be available to patients with COPD, even though some participants doubted whether smokers would look for this information. 

You don’t want to know; you feel that you are getting worse, and at a given moment with every cigarette I lighted I needed to lie down for 30 minutes. But you know, you just don’t want to know. 

Nevertheless, it was still considered important to have this information on the website.

Well, if you go toward the oxygen treatment, then you must stop smoking otherwise it is not allowed. 

Yes, it is very important to have this information available: repeat, keep repeating

Participants acknowledged that the website provided information about smoking cessation and referred smokers to smoking cessation products if they wanted to quit. However, participants felt it would have been better to refer these cases to a GP instead of commercial products. Although other participants still thought that providing this information was relevant, it was acknowledged that it should only be presented as an option for smoking cessation. 

They want to sell things and I want information. 

#### BMI Tool

The website included a BMI calculation tool that could be found under the heading “calculation tools.” However, several participants did not know what “calculation tool” meant, so it was not clear to them where the BMI tool could be found. Participants also had mixed opinions about the tool. On the one hand, some participants stated that they would never use it.

I cannot imagine that people will fill this in regularly. Not really. Certainly not if you don't feel well.

On the other hand, some participants thought that its inclusion was important. 

I know it is important because the pulmonologist puts you on a scale at set times. And he also wants to measure … to see how heavy you are. And once a year he will also do the tests of your lungs; so, it is part of it

the hospital visit

## Discussion

### Principal Findings

The overall aim of the study was to obtain information from elderly patients with COPD and asthma regarding a PWP. This cross-sectional qualitative study revealed that patients were generally positive about the website and its content. Most viewed the asthma and COPD-related information and the medication overview as valuable inclusions, particularly where it enabled them to share key information with other health care providers. However, the comments were not universally positive. Many experienced difficulties with navigation, particularly when opening a new tab and returning to the original page, and the website’s layout was criticized because it was considered difficult to use by patients with visual impairments. Although the potentially favorable role of monitoring was acknowledged, this was only considered relevant if it was simultaneously monitored by health care professionals as part of a treatment and follow-up plan. Surprisingly, participants raised few concerns about safety or privacy. The responses of participants were quite varied, which stresses the need for personalized solutions, especially among those with poor digital literacy. An easy-to-use “settings menu” can help to configure the application according to the user’s needs.

### Comparison With Existing Literature

#### General Attitudes and Safety Concerns

Consistent with our research, other authors have reported that elderly people tend to have positive attitudes toward digital health. The available literature supports the effectiveness of including disease-related information, and medication overviews providing the information are customized to provide relevant information to each patient [13]. Interestingly, however, while prior research has shown that trust in the security of a PWP is an important issue [21], our patients had no real concerns about privacy and safety. Some patients even mentioned that they did not like passwords and they would prefer an easier way to enter the website, with no mention of safety concerns. Although we should not take this to imply that appropriate safety measures can be overlooked, it may be that patients will accept lesser levels of security as a tradeoff for greater convenience. Participants suggested that it may be possible to use the Dutch governmental authorization system with 2-factor authentication involving a password and an SMS text message code. This method is often used in health care settings and is generally considered to be safe, but as we noted, some patients did not have access to a mobile phone. Developing the existing system to include alternative methods of authentication could prove beneficial (eg, landline, email, or CAPTCHA [completely automated public Turing test to tell computers and humans apart] systems). According to the General Data Protection Regulation (GDPR), appropriate data security is obligatory, and according to the Dutch Data Protection Authority, medical data need to be secured with 2-factor authentication. In order to provide elderly people access to their medical data, 2-factor authentication without a smartphone would be a wise approach. Two-factor authentication with an email address is more feasible, considering the fact that patients using digital health applications already have an email address.

#### Website Layout and Accessibility

Our patients tended to have difficulties in navigating the website. This was broadly consistent with the findings of Vancea et al [6], even though they reported that navigation skills for mobile devices with low complexity were comparable between younger and older populations. Indeed, many of our patients stated that they would like the website to be less complex, as supported by earlier research [21]. Compounding the lack of a clear user interface, many also noted the potential for limited vision to affect usage. Kuerbis et al [22] provided an excellent overview of digital health design considerations for elderly people, and in it, they stressed the importance of having a clear layout that could be read by people with poor vision. They also advocated using navigation aids and less complicated menus, and avoiding the need to open new tabs or windows, which again were consistent with our findings.

Based on the findings in this study, we recommend that the advice of Kuerbis et al [22] be implemented in future digital health designs for elderly people. Moreover, improving the contrast of text areas, increasing the sharpness and size of text, and including less information and clearer navigation on each page could certainly enhance readability. Given that most patients in our study did not know that they could enlarge the letters in the browser, simple notes could be included on the homepage or scalability could be better integrated into the website. For example, users who are not IT literate and have poor vision may not have their desktop and browser set to an appropriate magnification level. Websites using CSS (Cascading Style Sheets) often rely on “em” as the unit to upscale a base font, and we could improve accessibility by changing this to be larger in “px” units, with sans serif and high contrast font. Coupling these changes with text that is in a single column with multiple short paragraphs may negate many of the concerns that were raised by our participants. An increasing number of websites have adapted their layout to enhance usability for elderly people. For example, the Patient Access website [23] developed by the NHS (National Health Service) for primary care patients in the United Kingdom has a clear layout and an instant technical support option for users. 

#### Need for Support in the Use of Digital Health

Most of the elderly population in the Netherlands has internet access, but despite this, many participants in this study lacked the basic internet skills needed to navigate the website. In these cases, providing guidance or even training on digital health use could help to improve access and uptake. A Dutch study on older patients with cardiovascular disease showed that a coach helped patients to use an interactive self-management internet platform [24]. Informal IT support by peers or health care workers can also help elderly people overcome a lack of computer skills. We must acknowledge that this can threaten the privacy and autonomy of elderly users of digital health by potentially exposing confidential personal health data [6]. However, despite the apparent lack of basic internet skills, our participants did not mention the need for support or assistance with the website, which we thought could be due to the perception that digital health information is private, leading to a reluctance to seek support from family members, friends, or health care professionals. Nevertheless, we think that appropriately designed systems could circumvent the concerns. For example, using test accounts, giving proper tutorials [21], and ensuring that users receive training [22] can improve digital health usage, especially if that training is tailored to the needs of elderly people [25]. Overall, training can improve digital health literacy and navigation skills [7], and we believe that suitable courses could be implemented.

#### Need for Greater Involvement by Health Care Providers 

If digital health innovations are to be trusted and used, they need health care providers to advocate their use and refer patients to use them [6]. It was notable that participants wanted health care providers to be involved in monitoring the severity of asthma and COPD if they were using the questionnaire on the website. Several patients even mentioned that they would not use the questionnaires unless they were considered a necessary part of the assessment by their health care providers. This is supported by prior research showing that health care providers who promote the engagement of elderly people in mobile health can reduce digital health disparities in this group [26].

#### Need to Remove Barriers to Digital Health Use

Several models have been used to predict technology acceptance by elderly people, including the MOLD-US framework and the Senior Technology Acceptance Model. The MOLD-US framework provides a useful overview of the barriers faced by elderly people when using mobile health apps [16]. It shows that the usefulness and usability of mobile health apps for older adults may be affected by age-related physical function, perceptive and cognitive limitations, and motivations, including self-confidence and IT literacy. According to a Senior Technology Acceptance Model validation study, personal and environmental facilitators were more strongly related to usage behavior than either usefulness or ease of use. Relevant personal factors were age, education, digital health self-efficacy, anxiety, and health deficiencies, while relevant environmental facilitators were accessibility, assistance, and guidance [12].

Elsewhere, Latilupe et al [27] reported that motivation, negative attitude, trust, and lack of appropriate technology were the greatest predictors of acceptance. While personal factors like age and education are not modifiable, factors like anxiety, trust, self-efficacy, accessibility, and guidance can be modified. Indeed, stress and anxiety are important barriers to digital health acceptance. Moreover, using IT apps can enhance self-esteem and self-efficacy in elderly people by giving them access to more information and a sense of empowerment [25].

By removing the many barriers to digital health use and by reinforcing the facilitators, we can move toward systems that are of maximum utility to elderly patients. This is very important since they can benefit most from digital health–related self-management support due to the high prevalence of chronic diseases in this group.

### Strengths and Limitations 

The main strengths of this study were the qualitative design that allowed for free discussion, the inclusion of only users with no experience of the site, the ability of users to test the website before the focus groups, and the use of a broad interview schedule with questions about content and design. By having no exclusion criteria, we were also able to deliver a real-life cohort that included patients with comorbidities. These features ensured a more balanced assessment. However, there were also several limitations. First, we did not evaluate the effectiveness of the PWP. In research by Hallensleben et al [28], which provided an overview of Dutch digital health apps and websites for COPD, they concluded that there was a greater need for more research on the effectiveness of digital health strategies. Given that the clinical effectiveness of PWPs remains unclear, we must assess this in future studies. Second, the population was biased toward motivated patients with more positive attitudes to digital health topics owing to our cohort. This was compounded by only including 2 small focus groups that were not triangulated. Due to the nature of the study, patients with severe shortness of breath would not have been able to participate. Therefore, the participants with COPD might not be representative of the whole COPD population. Third, we only tested 1 website, only performed the assessment in the Netherlands (where a greater proportion of the population is online compared with other countries), and only considered patients with asthma and COPD. Although these factors limit the generalizability of our data to other populations, we are satisfied that they reflect many Western societies and have relevance to populations with chronic NCDs. Finally, it was notable that other key stakeholders, such as health care professionals, were not involved. Future research will need to address these issues. 

### Conclusion and Future Implications

We evaluated the first impressions of elderly patients with asthma and COPD regarding a pharmacy PWP. Our results are strongly in agreement with those of previous research advocating the need to take into account specific difficulties faced by elderly people, such as poor vision or an inability to navigate websites. PWPs certainly need to include core information about disease, medication, and self-management, because information provision was considered to be the most important aspect by patients. Training can also help to overcome the digital divide, which can be facilitated by the government reducing the financial barriers to access and support. However, it was also evident that PWPs should be considered part of a formal treatment and monitoring plan that is used by patients and doctors alike. In short, PWPs must be designed in such a way that they are simple, easy to use, and self-contained, while ensuring that there is high uptake among practitioners to use them as a management aid. The training and design should be personalized toward the specific barriers of individual users. Patients will then be able to access relevant information and keep a record of their management, with motivation strengthened by the tacit acknowledgment that clinicians value this input.

## Abbreviations

COPD: chronic obstructive pulmonary disease
GP: general practitioner
NCD: noncommunicable disease
PWP: patient web portal

## Data Availability

The data sets generated during or analyzed during the study are available from the corresponding author on reasonable request.
